# Suitability of Anodic Porous Alumina as a Passive Radiative Cooler: An In-Depth Study

**DOI:** 10.1021/acsaom.3c00216

**Published:** 2023-08-15

**Authors:** Alba Díaz-Lobo, Marisol Martin-Gonzalez, Ángel Morales-Sabio, Cristina V. Manzano

**Affiliations:** † Instituto de Micro y Nanotecnología, IMN-CNM, CSIC (CEI UAM + CSIC), Isaac Newton, 8, E-28706 Tres Cantos, Madrid, Spain; ‡ Centro de Investigaciones Energéticas, Medioambientales y Tecnológicas (CIEMAT), Avda. Complutense, 22, E-28040 Madrid, Spain

**Keywords:** anodic aluminum oxide, alumina nanostructures, passive radiative cooling, solar reflectance, infrared emittance, cooling power

## Abstract

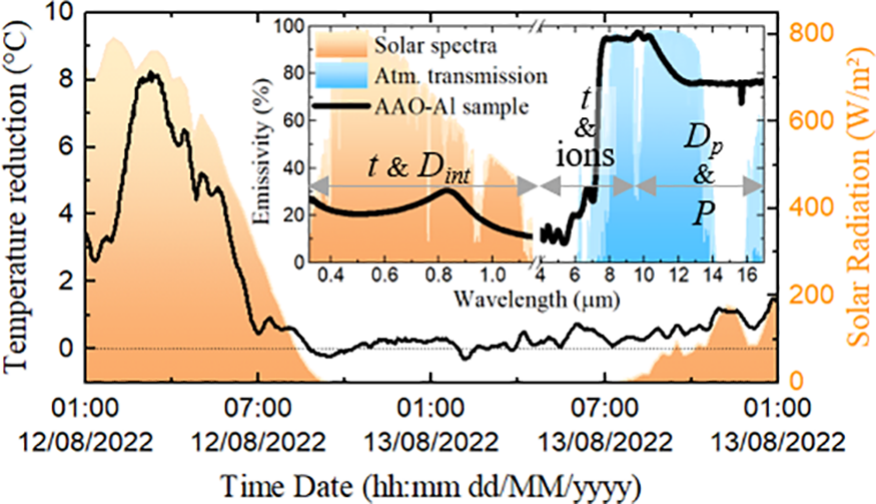

Passive radiative cooling technology has the potential to revolutionize the way of cooling buildings and devices, while also helping to reduce the carbon footprint and energy consumption. Pioneer works involving anodic aluminum oxide (AAO) nanostructures showed controversial results. In this work, we clarify how the morphological properties and chemical structure of AAO–Al samples affect their optical properties and their cooling performance. Changes in thickness, interpore distance, and porosity of the alumina layer, as well as the used counterions, significantly affect the cooling ability of the AAO–Al structure. We measure a maximum temperature reduction, Δ*T*, of 8.0 °C under direct sunlight on a summer day in Spain, coinciding with a calculated peak cooling power, *P*_cool_, of 175 W/m^2^, using an AAO–Al sample anodized in sulfuric acid, with 12 μm of AAO thickness and 10% of porosity. These results represent a significant improvement over previous studies, demonstrating the potential of AAO nanostructures to be used in thermal management applications.

## 1 Introduction

Global warming has led to higher temperatures and more frequent heatwaves, resulting in increased energy consumption for cooling, which accounts for a third of the total energy demand.^1,2^ Moreover, energy consumption due to air conditioners is expected to increase in the next decade.^3^ To tackle this issue and save energy, passive radiative cooling technology has emerged as an efficient solution.

Passive radiative cooling, traditionally used for nocturnal cooling,^4−6^ has potential for diurnal cooling^7,8^ when high solar reflectivity meets high thermal emittance, especially through the main atmospheric window (wavelengths between 8 and 13 μm). Therefore, developing a cooling technology which cools without any power input can make a significant difference in energy savings by reducing the use of air conditioners. Multiple applications^9−12^ can take advantage of this cooling method beyond building cooling,^13^ such as improvements in the thermal comfort of textiles for personal thermal management^14−18^ or improvements in the efficiency of electronic devices by avoiding overheating.^19,20^ Different approaches have been explored, such as multilayers,^7,21^ paints,^22,23^ photonic structures,^7,24,25^ porous^26−28^ or metallized polymers,^29^ polymer dielectric composites,^30^ and natural materials.^31,32^ The porous nanostructures have shown the best results,^33,34^ becoming an interesting characteristic to improve the cooling performance of a material. This has encouraged scientific research on porous anodic aluminum oxide (AAO) nanostructures, which is an amorphous material with an isotropic permittivity, a strong acoustic resonance absorption at the far IR (15–25 μm), and high transparency in the UV–vis–NIR range.^35^

AAO nanostructures showed their daytime cooling ability in 2019, with a solar reflectivity  of 94%, an IR emittance  of 90%, a cooling power (*P*_cool_) of 64 W/m^2^, and a temperature reduction (Δ*T*) of 2.6 °C.^35^ Contrary to this first demonstration, other AAO nanostructures did not achieve cooling,^36^ whereas combining those porous AAO with SiO_2_ nanoparticles resulted in a Δ*T* of 4.7 °C. A theoretical study reported that some morphological parameters could influence the  of the AAO nanostructures,^37^ the porosity and the alumina thickness standing out. Then, high-porosity AAO nanostructures achieved a *P*_cool_ of 71.0 W/m^2^ and a Δ*T* of 6.7 °C in flexible films.^38^ AAO nanostructures combined with a SiO_2_ coating and a Ti/Ag layer reached an estimated *P*_cool_ of 65.6 W/m^2^ and a Δ*T* of 6.1 °C.^39^ Finally, calculations for 6.5 μm AAO nanostructures over an Al substrate suggested a *P*_cool_ of 136 W/m^2^ but without experimental demonstration.^40^ Therefore, dissimilar conclusions appear related to AAO nanostructures, combining promising results with no cooling ability. These variations support, but do not explain, the influence of the morphology on the cooling performance. Table 1 summaries the details related to the aforementioned nanostructures’ fabrication and morphology [pore diameter (*D*_p_), interpore distance (*D*_int_), porosity (*P*), and alumina thickness (*t*)], along with the optical properties ( and ) and the reported cooler’s details (structure, *P*_cool_, and Δ*T*).

**Table 1:** Summary of the Anodization Conditions and the Morphological Parameters of the AAO Nanostructures, along with the Reported Cooler’s Performance Details Published in the Literature

anodization conditions	*D*_p_ (nm)	*D*_int_ (nm)	*P* (%)	*t* (μm)	*R*_sol_ (%)	ε_IR_ (%)	cooler structure	*P*_cool_ (W/m^2^)	Δ*T* (°C)	ref
oxalic acid	280	340	82	56.8	99.4	90	AAO + AAO glue + Al	64	2.6	(35)
commercial	350			10	95	5 (10 μm)	AAO	0	0	(36)
0.3 M oxalic acid, 40 V, 5 °C	60	100		20	95	98 (10 μm)	AAO NTs + SiO_2_ + Ag + PDMS	71	6.7	(38)
0.1 M phosphoric acid, 195 V, 0 °C	400	500		50	86	96	AAO + SiO_2_ + Ti/Ag	65.6	6.1	(39)
0.3 M sulfuric acid, 25 V, 0 °C	15	600	5.7	6.5	98	88 (8–13 μm)	AAO–Al	136		(40)
						99 (10 μm)				

The cooling performance of the AAO nanostructures is expected to depend on the morphology as well as on the fabrication conditions, and there are multiple possibilities related to AAO nanostructures. However, as of today, there is not a clear study to understand how all the alumina morphological parameters influence the AAO nanostructures’ optical response and, as a consequence, which are the best morphologies to develop passive radiative coolers. A proper selection can make a huge difference in a cooler’s structure, boosting or limiting cooling. Therefore, in this work we study different electrolytes (phosphoric acid, oxalic acid, sulfuric acid, and ethylene glycol containing sulfuric acid) to carry out the anodization process, which provides wide ranges for *D*_p_ and *D*_int_ to tune the morphological properties of the AAO nanostructures. We perform a complete morphological and optical characterization of the nanostructured AAO on Al foils. The morphological study includes the pore’s arrangement order grade, *t*, *D*_p_, *D*_int_, *P*, and the presence of different counterions, incorporated from the different electrolytes, along with the optical characterization analyses  as well as . Also, we carry out calculations of *P*_cool_ and Δ*T* at nighttime and daytime. Furthermore, we measure experimentally the temperature reduction achieved by the AAO–Al samples under different weather conditions. Hence, we study exhaustively the influence of the morphological properties, the chemical composition of AAO nanostructures, and their effect on the optical properties and on the cooling performance to ensure a correct choice in future works.

## 2 Materials and Methods

### 2.1 Fabrication of the Nanostructured AAO on Al Foils

The fabrication of the AAO nanostructures follows a standard two-step anodization process, detailed in previous work.^41^Table 2 shows the specific anodization conditions. These anodization conditions (applied voltage, anodization temperature, and first anodization time) have been chosen to reach self-ordering and self-assembly ranges, as it was previously reported.^42−44^

**Table 2:** Anodization Conditions of the AAO–Al Samples

electrolyte	applied voltage (V)	anodization temperature (°C)	first anodization time (h)
1 wt % phosphoric acid + 0.01 M Al oxalate	200	1	6
0.3 M oxalic acid	40	3	24
0.3 M sulfuric acid	25	0	24
50 wt % ethylene glycol + 10 wt % sulfuric acid	19	0	24

The resulting AAO–Al samples were chemically etched in phosphoric acid (5 wt %, 85% Sigma-Aldrich) at 30 °C, varying the time to enlarge the *D*_p_ gradually.

### 2.2 Characterization of the Nanostructured AAO on Al Foils

Morphological characterization was conducted using high-resolution field emission scanning electron microscopy (FE-SEM, FEI VERIOS 460) with a 2 kV accelerating voltage. Top view and cross-sectional images were taken and digitally processed using XnView and ImageJ software. First, the reproducibility of the fabrication process was validated by analyzing 4 AAO–Al using the same anodization condition. Each sample was studied by FE-SEM to measure the AAO thickness in multiples regions of the sample at least four times, as well as the pore diameter and the interpore distance along the AAO surface, considering thousands of pores during the statistical analysis (further details are shown in the Supporting Information). Then, this article has focused on the mean value of each parameter together with its standard deviation for each type of AAO–Al sample because of the high reproducibility during the fabrication process. The errors of derived parameters, such as porosity, were calculated by error propagation. The  of the AAO–Al samples was measured using a Specord 210 Plus UV–vis spectrophotometer with an accessory Spectralon (PTFE) integrating sphere, ranging from 320 to 1150 nm. To characterize the optical behavior in the mid-IR, from 5 to 17 μm, a Fourier transform infrared (FT-IR) spectrophotometer of PerkinElmer (Frontier) was used, equipped with a 75 mm diameter integrating gold sphere to collect both specular and diffuse reflectance components. Then, was obtained by 1 – *R* since the Al substrate is an optically opaque layer. The solar irradiance data corresponding with the background AM 1.5 G spectrum was obtained from the National Renewable Energy Laboratory Web site,^45^ and the atmosphere transmission data was available at the Gemini Observatory Web site.^46^

### 2.3 Experimental Setup for Outdoor Measurements of the Passive Radiative Cooling Performance

**Figure 1:**
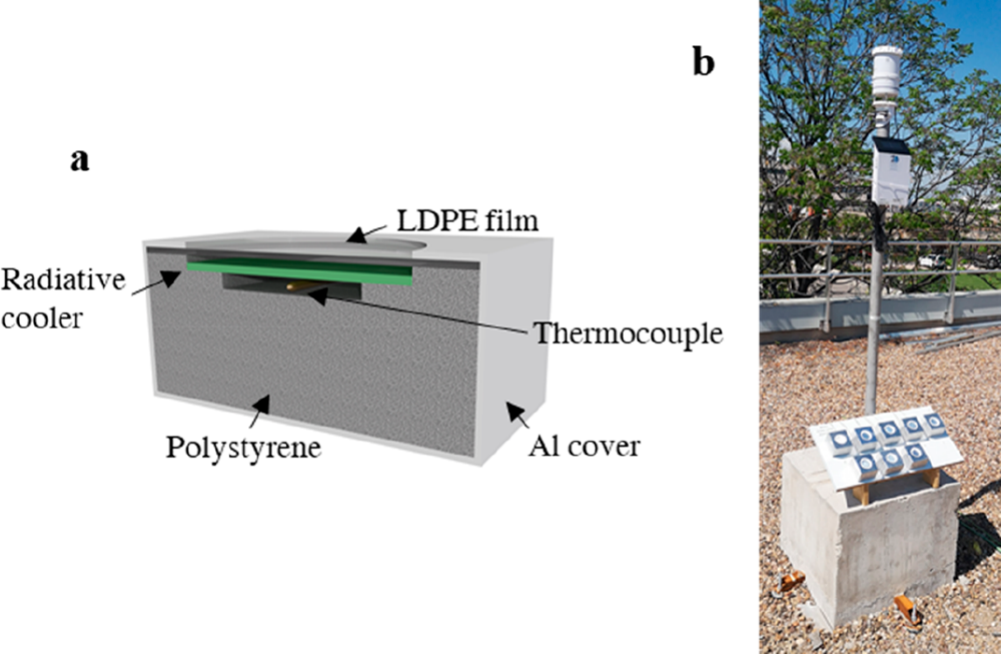
(a) Schematic diagram of the experimental system and (b) setup placed on the rooftop, nearby the weather station in Madrid, Spain.

The maximum *P*_cool_ has been calculated following eqs S2–S7 in the Supporting Information, analogously to ref (7). The performance of the AAO–Al samples as passive radiative coolers has been characterized using the experimental setup shown in Figure 1a and b, placed on the building’s rooftop.

The setup consists of 7.5 cm × 7.5 cm × 5 cm polystyrene foam blocks with a cylindrical groove of 0.5 cm and a diameter of 3.5 cm to act as thermal insulator where the passive radiative cooler is placed. The foam is covered by an aluminum foil to reflect solar irradiance and keep the block from overheating. To reduce the convection, a low-density polyethylene (LDPE) film of 25 μm (Goodfellow) seals the foam, improving the thermal insulation. The setup is located over a wooden frame with a 30° tilt to maximize the exposure of the coolers to solar radiation during the daytime. K-type thermocouples are in contact with the bottom face of the coolers to measure the real-time temperature variations, which are recorded by a Huato (S220-T8) data logger. A weather station of the METER Group (ATMOS41) is placed nearby the experimental setup to record solar radiation, air temperature, and relative humidity.

## 3 Results and Discussion

### 3.1 Morphological Characterization

**Figure 2:**
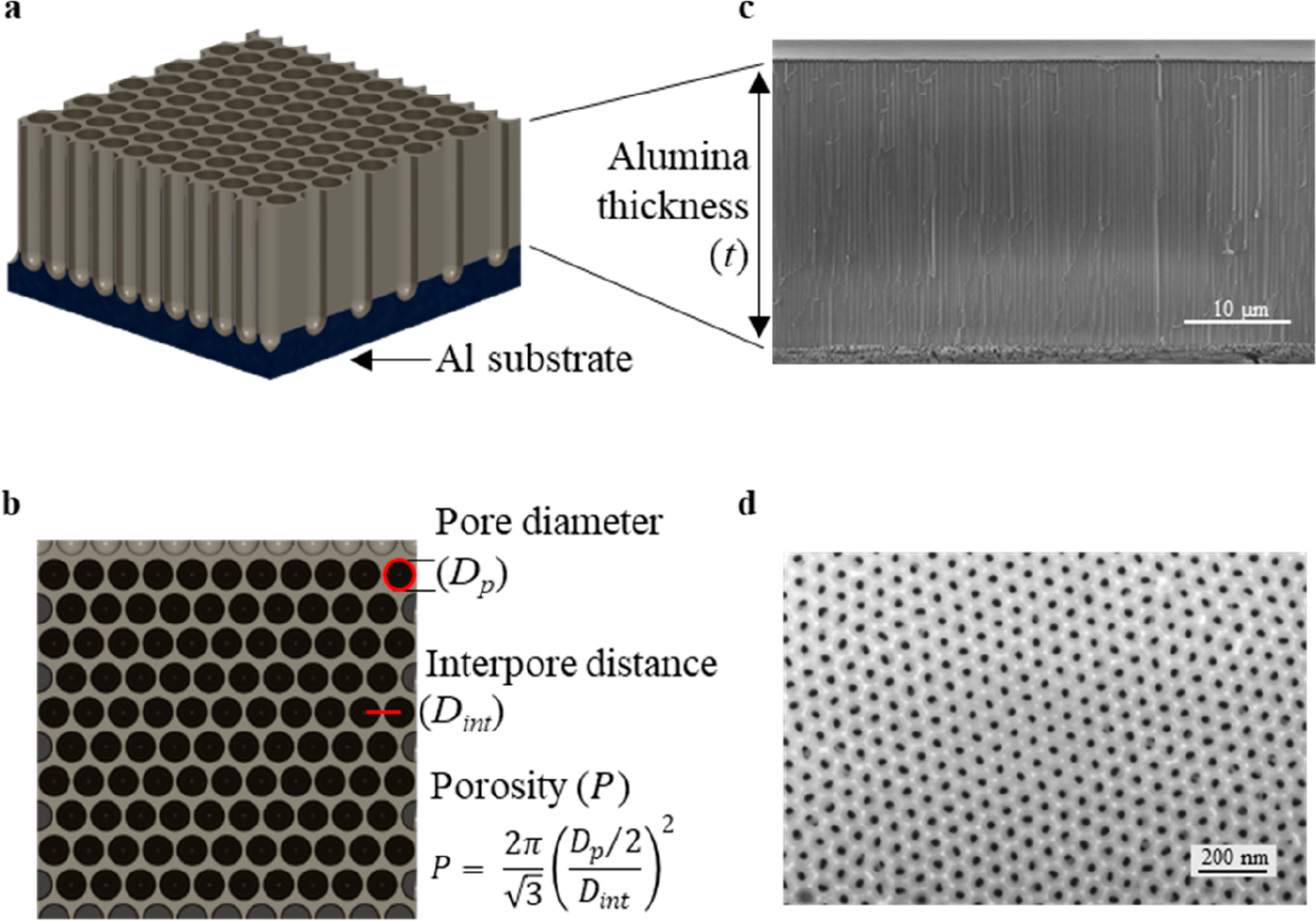
(a) Schematic of the nanostructure of the AAO on the Al substrate, (b) top view for high-ordered AAO nanostructures, and examples of real SEM images for (c) cross section and (d) top view.

There is a closed relationship between the optical properties and the morphology of the AAO nanostructures.^47^ Therefore, the Supporting Information shows a detailed morphological characterization of the AAO–Al samples, considering the pore’s arrangement order grade, *t*, *D*_int_, *D*_p_, and *P*. Figure 2 shows a schematic of the AAO–Al samples nanostructure.

### 3.2 Optical Characterization

The study of  and  of the AAO–Al samples gives special attention to the influence of the morphological parameters and the chemical composition of the AAO nanostructures.

The pore’s arrangement order grade has no effect on  (see Figure S4 in the Supporting Information) owing to the high AAO transparency. However, as Figure S4 shows,  increases about 5% after the second anodization, if the one-anodized AAO–Al sample showed  < 96%, because of the higher order grade, which meets the emergence of ordered domains and improves the AAO’s surface quality (independently of the electrolyte). Then, the fabrication of all the AAO–Al samples follows a two-step anodization process.

**Figure 3:**
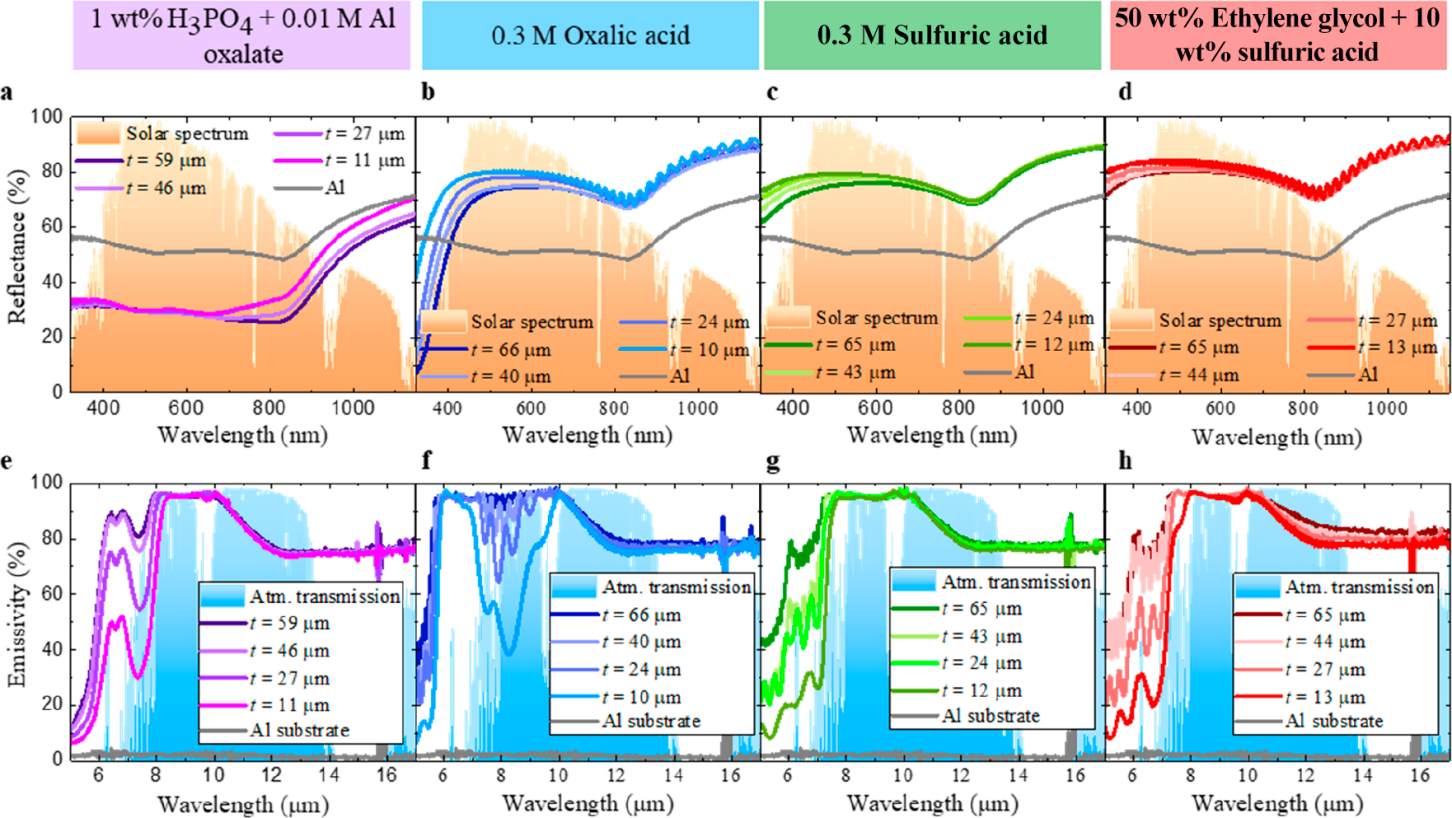
and  spectra as a function of alumina’s thickness for AAO–Al samples anodized in (a, e) phosphoric acid, (b, f) oxalic acid, (c, g) sulfuric acid, and (d, h) ethylene glycol containing sulfuric acid.

To study the effect of varying the alumina thickness (*t*), Figure 3 shows the  and  measurements, grouped by common electrolyte to minimize noise due to *D*_int_, *D*_p_, or *P* variations.

There is a notable local minimum at 850 nm in all the  (Figure 3a–d) spectra, associated with the interband transition of the Al substrate.^48^ Smooth influence of AAO thickness appears for AAO–Al samples anodized in phosphoric acid (Figure 3a) from 650 to 1150 nm and in oxalic acid, sulfuric acid, and ethylene glycol containing sulfuric acid (Figure 3b–d) from 320 to 700 nm. A thinner AAO layer corresponds with higher  (≈10%), reaching the maximum when *t* = 12 μm in all the electrolytes. Figure S5a–d in the Supporting Information shows thinner AAO layers, between 3 and 6 μm, but there are no improvements in . This behavior is consistent with previous studies from our group for oxalic, sulfuric, and ethylene glycol containing sulfuric acid,^41^ in which the maximum  increases as *t* and *D*_int_ decrease. The behavior for the different electrolytes is similar in terms of the influence of the alumina thickness on  (see Figure 3e–h): a thinner AAO layer means lower radiator volume between 5 and 8 μm; hence,  decreases in this range from 75 to 90%, 30 to 50%, and even to 25% for AAO thickness of 65 μm, 12 μm, and thinner layers, respectively (see Figure S5e–h in the Supporting Information). In the case of oxalic acid (Figure 3f), the behavior is analogous up to 10 μm.

**Figure 4:**
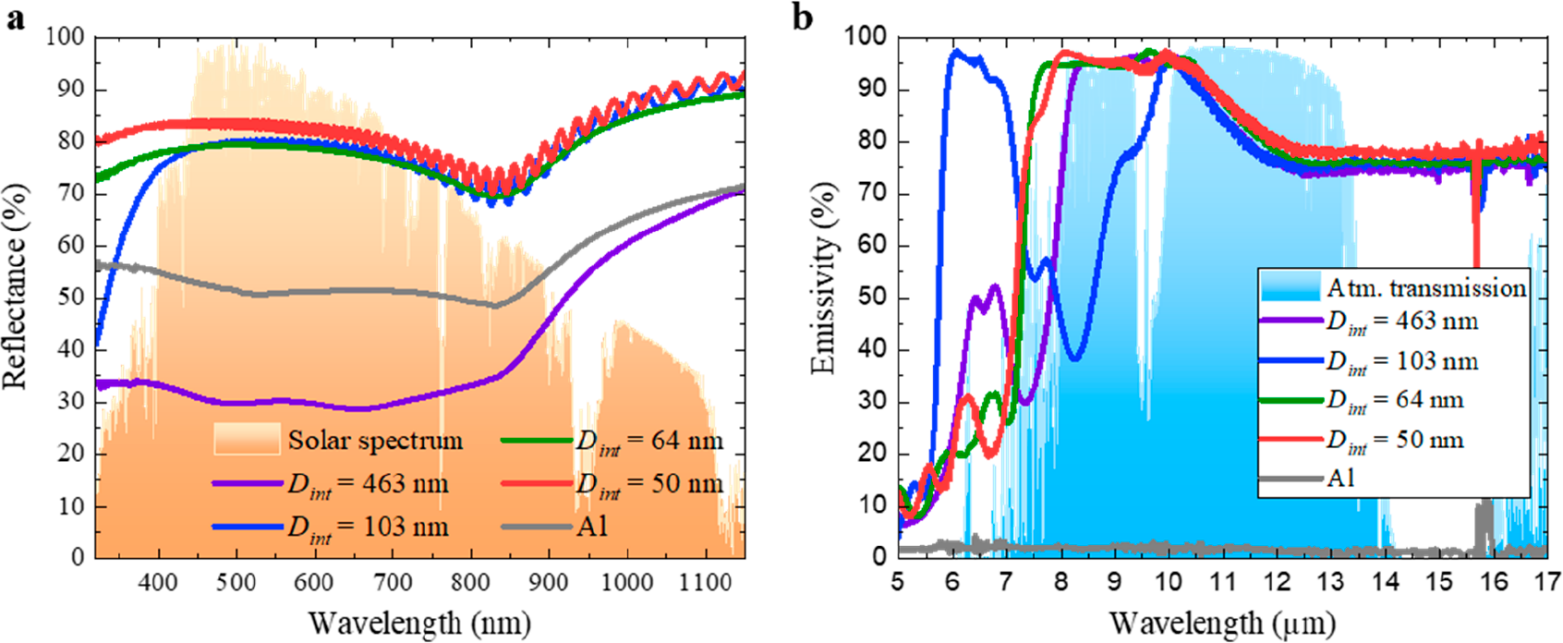
(a)  and (b)  spectra for 12 μm thick AAO–Al samples as a function of the used electrolyte.

To study the effect of the interpore distance (*D*_int_), Figure 4 shows a comparison of AAO–Al samples with the same *t* (12 μm) and anodized in the different electrolytes.

**Figure 5:**
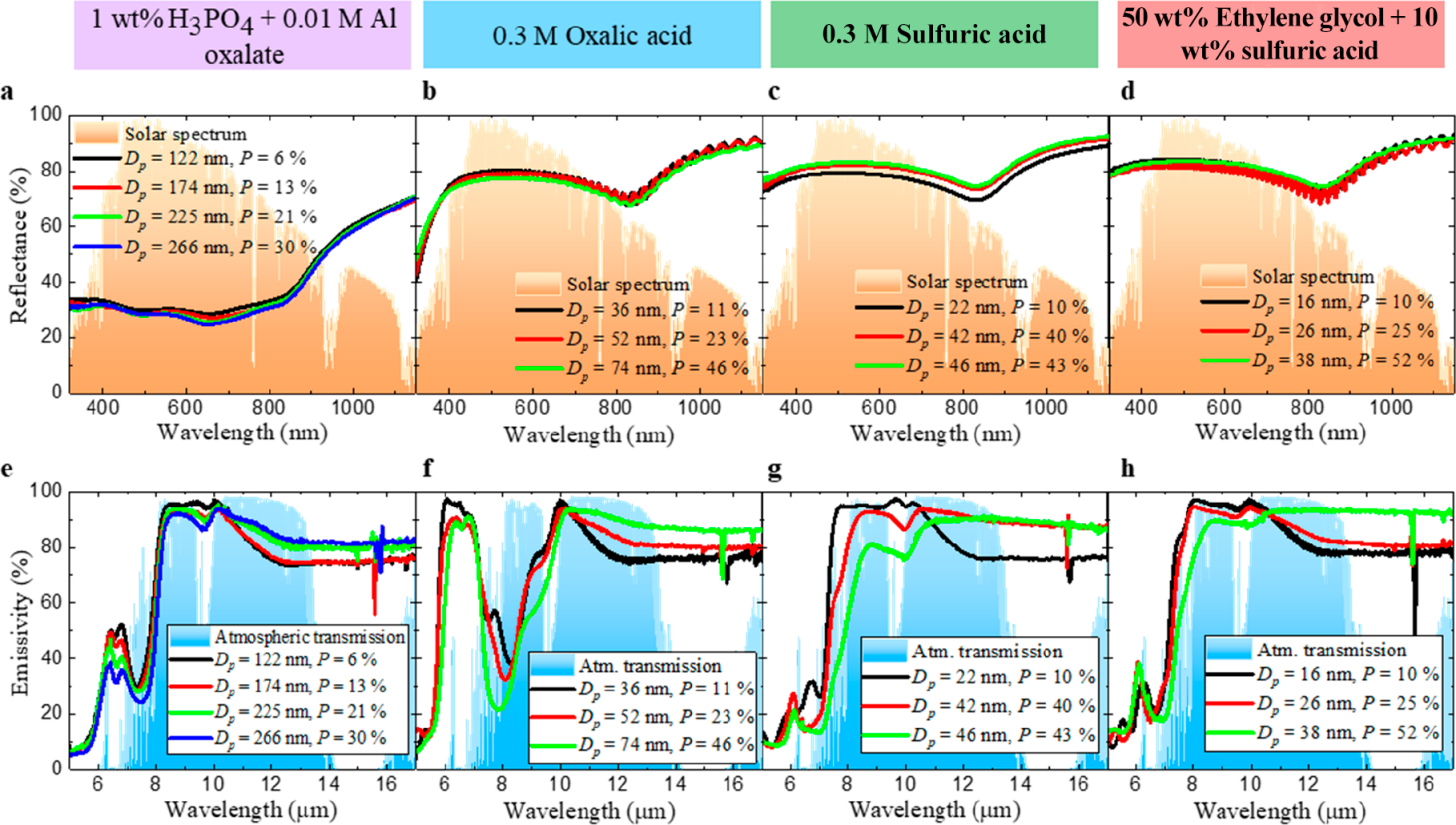
and  spectra evolution for progressive chemical etching for AAO–Al samples anodized in (a, e) phosphoric acid, (b, f) oxalic acid, (c, g) sulfuric acid, and (d, h) ethylene glycol containing sulfuric acid.

When the anodization is carried out in phosphoric acid (*D*_int_ = 463 nm), the  (see Figure 4a) between 320 and 850 nm is around 35%, which is nearly 20% less than that for the bare Al substrate. This behavior is due to the larger mean *D*_int_.^41^ When anodization is performed in oxalic acid (*D*_int_ = 103 nm), sulfuric acid (*D*_int_ = 64 nm), or ethylene glycol containing sulfuric acid (*D*_int_ = 50 nm), the reduction of mean *D*_int_ allows for higher  than for the bare Al substrate. The  values are 90%, 89%, and 91% for oxalic acid, sulfuric acid, and ethylene glycol containing sulfuric acid, respectively. Therefore, lower *D*_int_ will result in higher  values. The other AAO thicknesses show this same tendency. Depending on the electrolyte, different  shapes appear at wavelengths (λ) between 5 and 10 μm. Furthermore, at 10 μm  ∼ 97% and then decreases at 12.5 μm, showing different values of  (75–80%). There are multiple possibilities to explain these behaviors, through the *D*_int_, *D*_p_, and/or *P*. Therefore, to isolate their effect, we perform chemical etching of *D*_p_ and *P* to enlarge them gradually, while *D*_int_ stays constant (see Figure 5) and *t* = 12 μm.

The final increment in *D*_p_ is over 200% for all the electrolytes (see Table S2 in the Supporting Information), and no meaningful change appears in  (see Figure 5a–d). Therefore, *D*_p_ and *P* have no effect on the optical response in the UV–vis–NIR range. With respect to , one can observe two opposing behaviors in every electrolyte (Figure 5e–h) depending on the wavelength (λ). For 5 μm < λ < 10 μm, the emissivity decreases between 5% and 15%, smoothing the curve’s shape. For 10 μm < λ < 17 μm,  increases from 75% to 96% (the saturation value) for *P* = 10% and *P* = 52%, respectively. Figure 5h shows this tendency for AAO–Al samples anodized in ethylene glycol containing sulfuric acid after a chemical etching of 21 min. This enhancement in  is due to the change in the complex refractive index caused by the *P* increment, which modifies both the refractive index and the extinction coefficient.^35,37^

**Figure 6:**
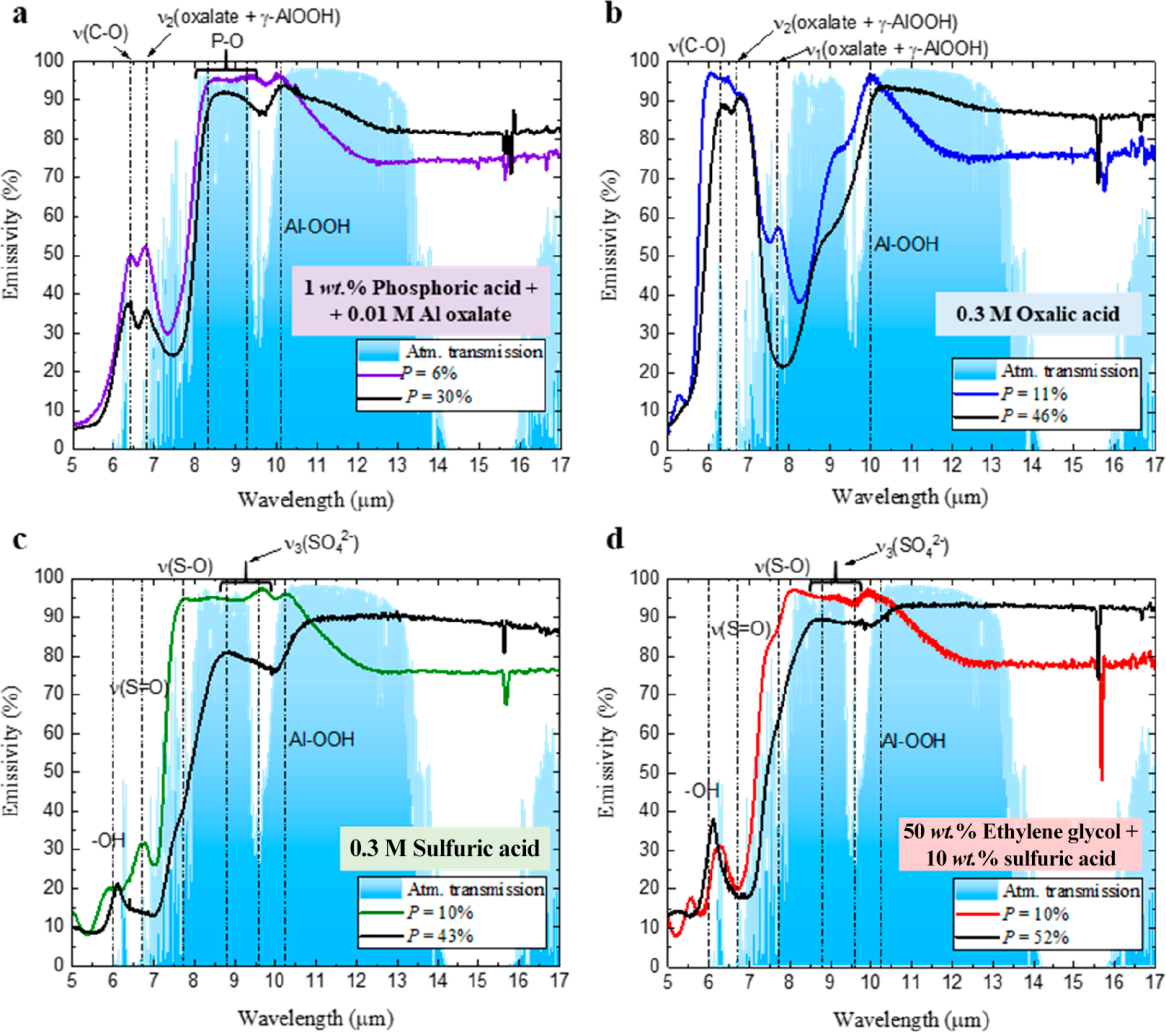
Identification of the main IR absorption bands from the specific incorporated ions during the anodization in (a) phosphoric acid, (b) oxalic acid, (c) sulfuric acid, and (d) ethylene glycol containing sulfuric acid.

To understand why  decreases between 5 and 10 μm when *D*_p_ enlarges, Figure 6 shows the identification of the IR absorption bands from the different impurities incorporated into the alumina wall during the anodization process.

Some bands are common in all the AAO–Al samples, like water, which results in hydrated alumina with Al–OOH bonds, whose vibrations go from 10 to 10.5 μm depending on the water proportion,^49,50^ or CO_2_ species incorporated from the surrounding environment, with a characteristic ν_1_ vibration mode around 5.6 μm.^51^ Other bands correspond to the specific ions from the different electrolytes incorporated into the AAO nanostructure.^52−55^ Thus, for phosphoric acid (Figure 6a) there is a P–O stretching between 8.3 and 9.3 μm.^53,56^ In addition, a broadband assigned to the ν(C–O) stretching mode appears at 6.4 μm, together with the vibration mode ν_2_(oxalate + γ-AlOOH) at 6.8 μm, due to the presence of Al oxalate in the phosphoric acid solution.^57,58^ For oxalic acid (Figure 6b), the ν(C–O) stretching mode also appears at 6.3 μm, together with the characteristic vibration mode from different oxalate–alumina combinations:^58^ ν_1_(oxalate + γ-AlOOH) at 7.7 μm and ν_2_(oxalate + γ-AlOOH) at 6.7 μm. With respect to the electrolytes containing sulfuric acid (Figure 6c–d), ν(S=O) and ν(S–O) show characteristic bands at 6.7 and 7.7 μm,^59^ respectively, and ν_3_(SO_4_^2–^) has a vibration mode from 8.8 to 9.6 μm.^60^ There are OH vibrations around 6 μm.^59^ The ethylene glycol contained in the electrolyte does not provide additional ions inside the AAO nanostructure when it is added to sulfuric acid electrolyte.^41^ It is worthy to note that the full set of IR absorption bands tunes the  shape between 5 and 10 μm. Moreover, the incorporated ions from the electrolyte create a gradual composition from the pore’s wall^42,54,61^ where their concentration is the highest, toward the outer alumina region (see Figure S6 in the Supporting Information). When *D*_p_ is enlarged by the chemical etchings, the alumina from the pore’s wall with the higher concentration is removed. Consequently, there is a reduction in the intensity related to the IR absorption bands of the ions in Figure 6 between 5 and 10 μm.

In conclusion, *t* and *D*_int_ influence , which reaches the maximum value (90%) for *t* = 12 μm and *D*_int_ = 52 nm, anodizing in ethylene glycol containing sulfuric acid. With respect to , for λ < 10 μm, the main contributions are the pore’s arrangement order grade, *t*, and the IR absorption bands of the counterions; and for λ > 10 μm, *P* is the principal influence. The AAO–Al samples with *t* = 12 μm, *P*_i_, and anodizing in sulfuric acid or ethylene glycol containing sulfuric acid achieve the best fit of the atmospheric window, in addition to corresponding with the maximum . Therefore, these AAO–Al samples with similar optical behavior are expected to show the best cooling performance under direct sunlight.

### 3.3 Calculations of Cooling Power

The analysis of the cooling power, *P*_cool_, and the temperature reduction, Δ*T*, based on the optical characterization shows significant variations at nighttime and daytime. Calculations of maximum *P*_cool_ follow eqs S2–S7 in the Supporting Information considering homogeneous emissivity, *T*_amb_ = 300 K, and a heat-transfer coefficient, *h*_CC_, of 12 W/m^2^·K for the following cases: the four electrolytes, 12 and 65 μm of alumina thickness, and initial (*P*_i_) and final porosity (*P*_f_) with *t* = 12 μm.

Table 3 shows the results of *P*_cool_ and Δ*T* during the nighttime. The value of *h*_*CC*_ has been chosen to simulate experimental conditions, but the effect of varying *h*_CC_ on the cooling performance properties is given in Figure S7 in the Supporting Information.

**Table 3:** Calculated Maximum *P*_cool_ and Δ*T* for Different AAO–Al Samples using *T*_amb_ = 300 K and *h*_CC_ = 12 W/m^2^·K during the Nighttime

		phosphoric acid + Al oxalate		oxalic acid		sulfuric acid		ethylene glycol + sulfuric acid	
		*P*_cool_ (W/m^2^)	Δ*T* (°C)	*P*_cool_ (W/m^2)^	Δ*T* (°C)	*P*_cool_ (W/m^2)^	Δ*T* (°C)	*P*_cool_ (W/m^2^)	Δ*T* (°C)
12 μm	*P*_i_	117	8	105	7	121	8	123	8
	*P*_f_	119	8	107	7	115	8	125	8
65 μm	*P*_i_	122	8	124	8	124	8	128	8

**Figure 7:**
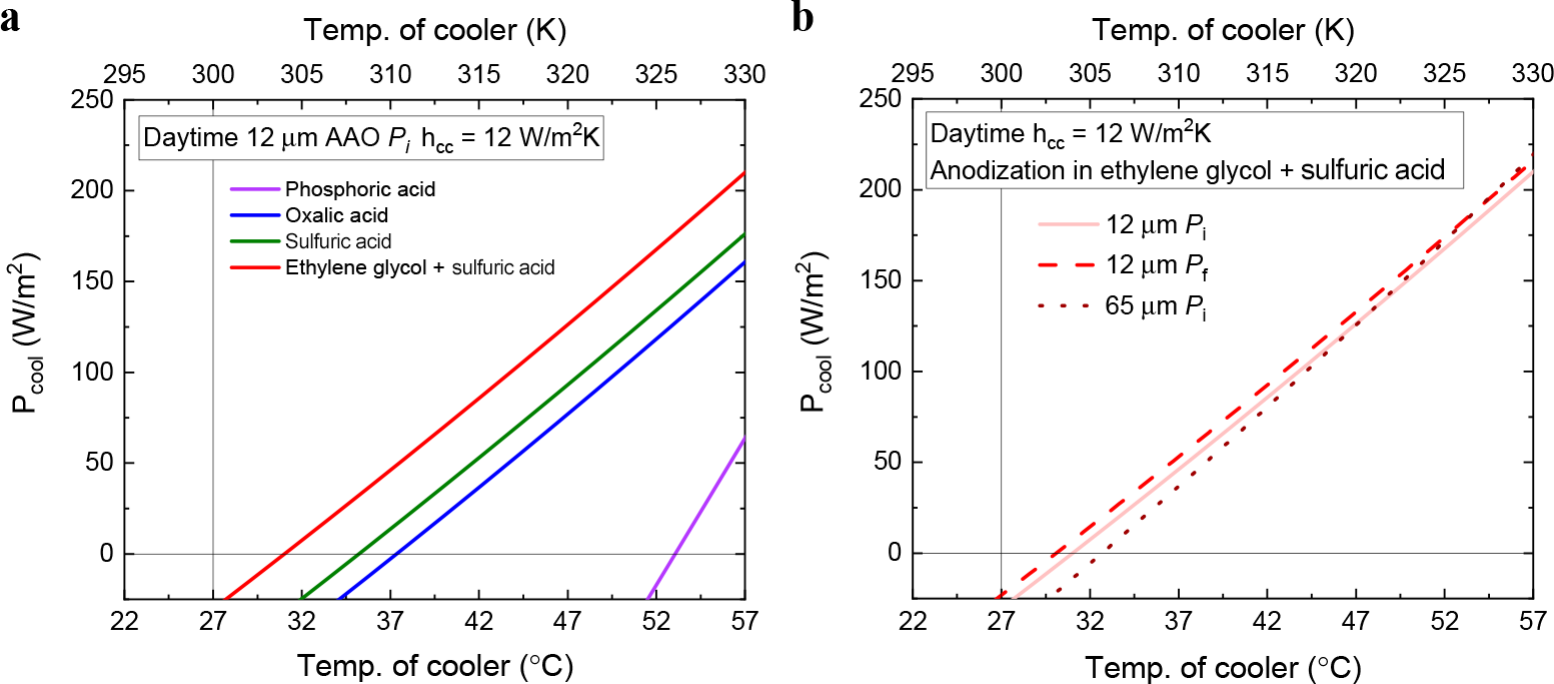
Calculation of *P*_cool_ and Δ*T* for AAO–Al samples at *T*_amb_ ≈ 27 °C (300 K) and *h*_CC_ = 12 W/m^2^·K under direct sunlight for (a) different electrolytes and (b) different AAO thicknesses and porosities.

All the considered AAO–Al samples show 128 W/m^2^ < *P*_cool_ < 105 W/m^2^, achieving the maximum *P*_cool_ for *t* = 65 μm, anodizing in ethylene glycol containing sulfuric acid, and the minimum *P*_cool_ for *t* = 12 μm anodized in oxalic acid. The different shapes of  (see Figure 4b) cause these variations. All the cases in Table 3 show 7 °C < Δ*T* < 8 °C; therefore, the whole set of AAO–Al samples is expected to show a similar performance during the nighttime. With respect to daytime, considering a typical temperature for a building’s rooftop (∼60 °C) on a summer day in Madrid as the initial cooler’s temperature, with *T*_amb_ ≈ 27 °C and *h*_CC_ = 12 W/m^2^·K, huge differences appear for *P*_cool_ depending on the electrolyte (see Figure 7a).

The AAO–Al samples at 57 °C show a maximum *P*_cool_ of 209, 176, 160, and 63 W/m^2^ when the electrolyte is ethylene glycol containing sulfuric acid, sulfuric acid, oxalic acid, and phosphoric acid, respectively. These wide ranges of *P*_cool_ result in Δ*T* of 26.0, 22.0, 19.5, and 4.0 °C for ethylene glycol containing sulfuric acid, sulfuric acid, oxalic acid, and phosphoric acid, respectively. During the daytime, the distinct behaviors of  (see Figure 4a) explain the different performances of the AAO–Al samples. Hence, the nanostructures anodized in ethylene glycol containing sulfuric acid show a greater potential for daytime passive radiative cooling at high temperatures, followed by the ones in sulfuric acid, whereas the nanostructures anodized in phosphoric acid seem to lack cooling ability during the daytime.

The expected variations of *P*_cool_ depending on *t* and *P* are shown in Figure 7b, considering the electrolyte with the best performance: ethylene glycol containing sulfuric acid. Comparing the values for *t* = 12 μm and *P*_i_ (*P*_cool_ = 209 W/m^2^ and Δ*T* = 26 °C), a thicker AAO layer results in an increment of 4.8% in *P*_cool_ but a minor Δ*T* = 24.5 °C. The increments of *P*_cool_ for a thicker AAO layer can be understood by the increment of  that occurs for 5 μm < λ < 8 μm (see Figure 3h). However, a higher  in these wavelengths does not result in a higher Δ*T* when exposed to direct sunlight; therefore, the thinner the AAO layer, the larger the Δ*T*. Higher porosity results in an increase of both *P*_cool_ and Δ*T*, reaching *P*_cool_ = 216 W/m^2^ and Δ*T* = 26.5 °C. The increment of *P*_cool_ is caused by the increment of  that occurs for higher porosity at λ > 10.5 μm (see Figure 5h), even when  decreases between 6 and 10.5 μm. Hence, the AAO–Al samples anodized in ethylene glycol containing sulfuric acid with *t* = 12 μm and *P*_f_ are expected to show the best performance under direct sunlight.

### 3.4 Outdoor Coolers’ Measurements

Furthermore, several cycles of measurements have been performed to study the cooling ability of the AAO–Al samples under different weather conditions.

**Figure 8:**
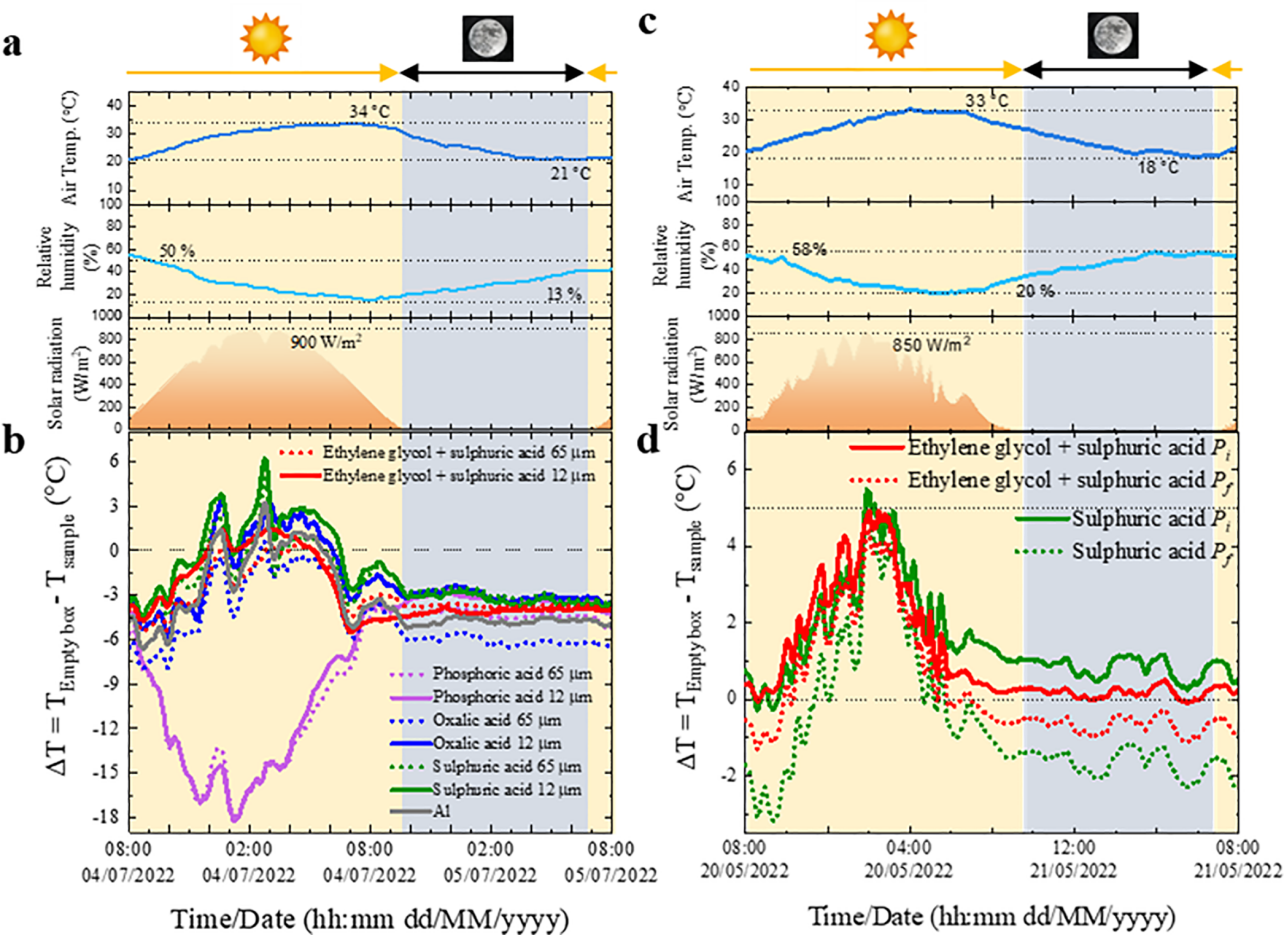
(a, c) Weather conditions during passive radiative cooling measurements of the AAO–Al samples with (b) different electrolytes and AAO thicknesses and (d) different porosities.

To verify the results calculated previously, we carried out several cycles of measurements of the AAO–Al samples, using the experimental setup shown in Materials and Methods, under different weather conditions in Madrid, Spain. The first cycle of measurements characterizes the AAO–Al samples comparing the different electrolytes and *t*. Figure 8a shows the details about the weather conditions. The air temperature varies between 34 °C (daytime) and 21 °C (nighttime), the relative humidity goes from 50% to 13%, and the solar irradiation reaches maximum values ≈880 W/m^2^.

Figure S8a in the Supporting Information shows the recorded temperatures, while Figure 8b shows Δ*T* achieved by the AAO–Al samples, considering an empty box as a reference. On July 4, 2022, the measurements focus on the AAO–Al samples anodized in different electrolytes with 12 and 65 μm in thickness and *P*_*i*_, together with a bare Al substrate. The temperature of the empty box varies from 20 to 50 °C, and so does the temperature of the coolers, following the temperature of the building’s rooftop at daytime. This is due to , which is not high enough to reflect the solar irradiance completely. However, this allows to achieve the maximum temperature reduction when it is the warmest, as well as a smooth reduction when temperatures are more comfortable. At night, the temperature of the empty box trends to 12 °C.

Beginning with daytime, huge differences in the cooling performance are found depending on the electrolyte, as the calculations point out. The AAO–Al samples anodized in phosphoric acid show a significant heating, reaching 18 °C above the temperature of the empty box under direct sunlight. In contrast with this heating ability, the rest of the AAO–Al samples show cooling ability under certain weather conditions, the maximum Δ*T* being 3.2, 6.1, and 1.4 °C for oxalic acid, sulfuric acid, and ethylene glycol containing sulfuric acid, respectively. The AAO–Al samples with a thicker AAO layer show a lower maximum Δ*T*, 0.6, 4.7, and 0.3 °C for oxalic acid, sulfuric acid, and ethylene glycol containing sulfuric acid, respectively. Therefore, according to the calculations, the thinnest AAO layer results in greater Δ*T* under direct sunlight. With respect to the nighttime, cooling is not achieved under these weather conditions (relative humidity of 22–40% and *T*_amb_ of 27–21 °C) in any case. However, one can appreciate variations in the behavior: temperature is maintained around 3 °C above the empty box’s temperature for phosphoric acid, oxalic acid, and sulfuric acid, whereas for ethylene glycol containing sulfuric acid the temperature is 1 °C higher when *t* = 12 μm. There are no significant changes when the AAO layer is thicker in ethylene glycol containing sulfuric acid and in sulfuric acid, but the temperature is maintained at around 6 and 4 °C above the empty box’s temperature for oxalic acid, and phosphoric acid, respectively.

The cycle of measurement on May 20, 2022 analyses the effect of *P* on the cooling performance, focusing on the anodization in sulfuric acid and ethylene glycol containing sulfuric acid with *t* = 12 μm. Figure 8c shows the details about the weather conditions. The air temperature varies between 33 °C (daytime) and 18 °C (nighttime), the relative humidity goes from 58% to 20%, and the solar irradiation reaches maximum values ≈870 W/m^2^. Figure S8b in the Supporting Information shows the recorded temperatures, while Figure 8d shows Δ*T* achieved by the AAO–Al samples. The temperature of the empty box varies from 15 to 50 °C during the daytime, and it trends to 15 °C at night. Beginning with daytime, the best performances, which correspond to the AAO–Al samples with *P*_i_, are Δ*T* = 5.5 and 4.9 °C anodized in sulfuric acid and ethylene glycol containing sulfuric acid, respectively, when the solar radiation is 848 W/m^2^, the relative humidity 26.7%, and *T*_amb_ = 30.2 °C. When the porosity is higher, the Δ*T* decreases to 5.1 and 4.3 °C for sulfuric acid and ethylene glycol containing sulfuric acid, respectively. With respect to the nighttime, the effect of *P* is critical to achieve cooling; the AAO–Al samples with *P*_i_ maintain a Δ*T* of 1 and 0.2 °C, whereas the ones with *P*_f_ show a heating of 1.6 and 0.7 °C for sulfuric acid and ethylene glycol containing sulfuric acid, respectively. Therefore, in contrast with the previous theoretical studies,^37,40^ enlarging *P* is not linked to improving the cooling performance of the AAO–Al samples because the chemical etching removes the incorporated ions from the chemical. These changes influence  (see Figure 6), particularly for the sulfuric acid anodization, as  decreases from 95% to 65–80% between 8 and 10 μm, whereas these losses for ethylene glycol containing sulfuric acid are limited to 80–90%. In conclusion, while the performance of the AAO–Al samples anodized in ethylene glycol containing sulfuric acid is higher under these weather conditions, and the best results are obtaining for AAO–Al samples anodized in sulfuric acid, with 12 μm of AAO layer and *P*_i_ of 10%.

**Figure 9:**
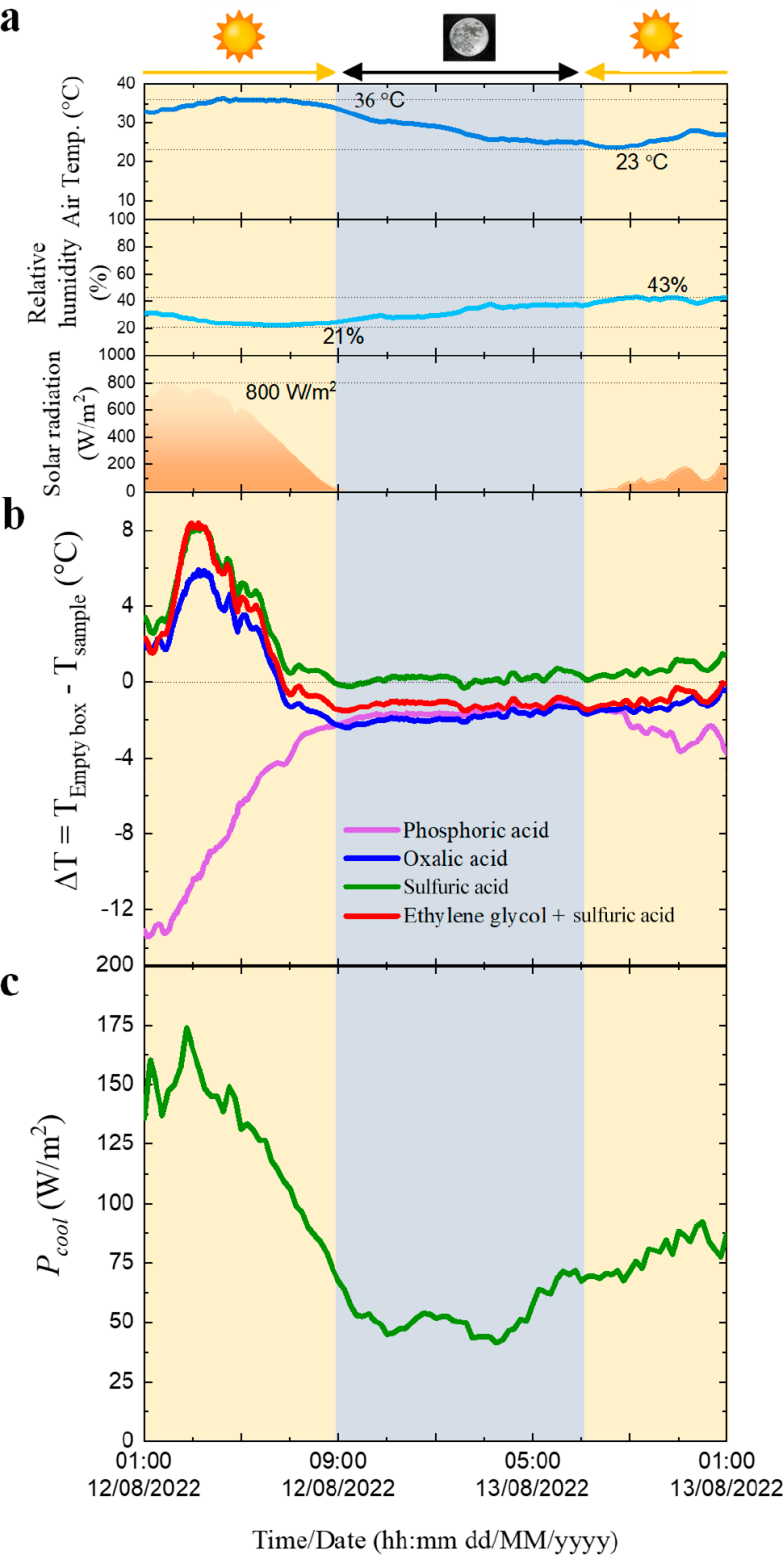
(a) Weather conditions during (b) the passive radiative cooling measurements of the AAO–Al samples for different electrolytes and (c) the calculated instantaneously *P*_cool_ for the best cooling performance.

It is worthy to note that the best performance is found in the same AAO–Al sample for the first and second measurements but with appreciable differences due to the weather conditions. Hence, the third cycle of measurements, on August 12, 2022, explores the variability of the AAO–Al samples (*t* = 12 μm and *P*_i_) with the weather conditions. Figure 9a shows the details about the weather conditions.

The air temperature varies between 36 °C (daytime) and 23 °C (nighttime), the relative humidity goes from 21% to 43%, and the solar irradiation reaches maximum values ≈800 W/m^2^. Figure S9 in the Supporting Information shows the recorded temperatures, while Figure 9b shows the Δ*T* achieved by the AAO–Al samples. In this cycle of measurements, the best measured performances during the daytime for the different electrolytes are under a solar radiation of 736 W/m^2^, relative humidity of 27.3%, and *T*_amb_ = 34.6 °C. Δ*T* is 5.4 , 8.0 , and 8.3 °C for oxalic acid, sulfuric acid, and ethylene glycol containing sulfuric acid, respectively. The AAO–Al sample anodized in phosphoric acid shows a maximum heating of 13 °C due to its low . This emphasizes the significance of utilizing the proper anodization acid in porous alumina, since it is demonstrated that the presence of PO_4_^3–^ within the alumina not only does not cool it but heats the material underneath, thus explaining the disparate results found in the scientific literature. During nighttime, only the nanostructure anodized in sulfuric acid can produce cooling, whereas the rest maintain a temperature ≈2 °C above the empty box’s temperature. The information about the weather conditions and the maximum Δ*T* achieved under direct sunlight for the AAO–Al samples anodized in sulfuric acid is summarized in Table 4 for the three performed cycles of measurements, highlighting the crucial role of the solar radiation in the daytime passive radiative cooling process: the maximum solar radiation (848 W/m^2^) corresponds to the worst cooling performance (Δ*T* = 5.5 °C) whereas the best cooling performance (Δ*T* = 8.0 °C) was achieved during the cycle with the minimum solar radiation (736 W/m^2^).

**Table 4:** Summary of the Maximum Δ*T* Obtained during the Daytime by the AAO–Al Samples with 12 μm AAO Thickness, *P*_i_, and Anodization in Sulfuric Acid under Different Weather Conditions

Δ*T*_cool_ (°C)	solar radiation (W/m^2^)	relative humidity (%)	air temperature (°C)
6.1	748	26.7	31.4
5.5	848	26.7	30.2
8.0	736	27.3	34.6

Figure 9c shows a more accurate calculation of *P*_cool_ for the best cooling performance of the AAO–Al samples with *t* = 12 μm and *P*_i_, using the recorded data from the outdoor measurements for air temperature and solar radiation during the entire cycle, considering *h*_CC_ = 12 W/m^2^·K. In these conditions, for a maximum value of Δ*T* of 8.0 °C, the calculated peak *P*_cool_ of the AAO–Al sample reaches a value of 175 W/m^2^. This value is much higher than the pioneering demonstration (Δ*T* = 2.6 °C and *P*_cool_ = 64 W/m^2^) reported by Fu et al.^35^ for AAO nanostructures anodized in oxalic acid with a high porosity of 82%, showing the great potential of these nanostructures as passive radiative coolers.

## 4 Conclusions

This study provides crucial insights into the tunable optical properties of nanostructured AAO on Al foils, which have a significant impact on the cooling ability of these materials. The alumina thickness and interpore distance are the most influential parameters for , which reached a maximum value of 90% with *t* = 12 μm and *D*_int_ = 52 nm, anodizing in ethylene glycol containing sulfuric acid. Moreover, there are two distinct regions for , with the most significant contributions coming from the alumina thickness and incorporated ions from the electrolyte for 5 μm < λ < 10 μm, while the porosity dominated at λ > 10 μm. The AAO layer of 12 μm, *P* = 10%, using electrolytes based on sulfuric acid, achieves the best fit for the atmospheric window with  ∼ 95% (7 μm < λ < 10 μm). The cooling ability of the AAO–Al samples showed considerable variability depending on the kind of counterions within the AAO; the one anodized in sulfuric acid, with *t* = 12 μm and *P* = 10%, consistently reaches the maximum Δ*T*. This research has experimentally demonstrated that increasing the porosity does not necessarily lead to improved cooling performance of the AAO–Al samples. Notably, a decrease of 8.0 °C was achieved with a solar radiation of 736 W/m^2^, relative humidity of 27.3%, and ambient temperature of 34.6 °C, corresponding to a calculated peak *P*_cool_ of 175 W/m^2^. These results significantly surpass those published previously for passive radiative coolers based on AAO nanostructures, indicating that these durable and lightweight AAO–Al samples have enormous potential as thermal management materials in various applications, including building cooling, smart windows, and improving thermal control in automobiles, thereby contributing to energy savings.

## Data Availability

All data are available in the main text and/or the Supporting Information.

## Supporting Information Available

SEM images, modification of pore diameters after chemical etchings, UV–vis–NIR reflectance and IR emissivity results, pore diameter distribution measurements, distribution of incorporated ions into the AAO structure, heat-transfer coefficients during cooling power calculations, outdoor temperature measurements, detailed morphological parameters values, gradual evolution of morphological parameters, and definitions of porosity and cooling power (PDF)
