# Supplementary material for: Suitability of Anodic Porous Alumina as a Passive Radiative Cooler: An In-Depth Study
Source: ACS Appl Opt Mater. 2023 Aug 15;2(6):980–90. doi: 10.1021/acsaom.3c00216 (PMC11220729; doi:10.1021/acsaom.3c00216)
Supplement: Supplementary file 1 — ot3c00216_si_001.pdf [file ot3c00216_si_001.pdf]

Supporting Information

Suitability of Anodic Porous Alumina as Passive Radiative Cooler: an in-depth study

Alba Díaz-Lobo[a], Marisol Martin-Gonzalez[a,], Ángel Morales-Sabio[b], Cristina V. Manzano[a]*

[a]Instituto de Micro y Nanotecnología, IMN-CNM, CSIC (CEI UAM+CSIC), Isaac Newton, 8, E-28706, Tres Cantos, Madrid, Spain

[b]Centro de Investigaciones Energéticas, Medioambientales y Tecnológicas (CIEMAT), Avda. Complutense, 22, E-28040, Madrid, Spain

Anodic aluminium oxide (AAO) nanostructures are produced by Al anodizing in an acidic medium[1, 2]. It is well-known that the pore's arrangement order grade is improved when a two-step anodization process is carried out, in comparison with the first anodization process[3].

To analyse the pore's arrangement, FE-SEM top view images after first and second anodization have been measured for the different electrolytes (see Figure S1).

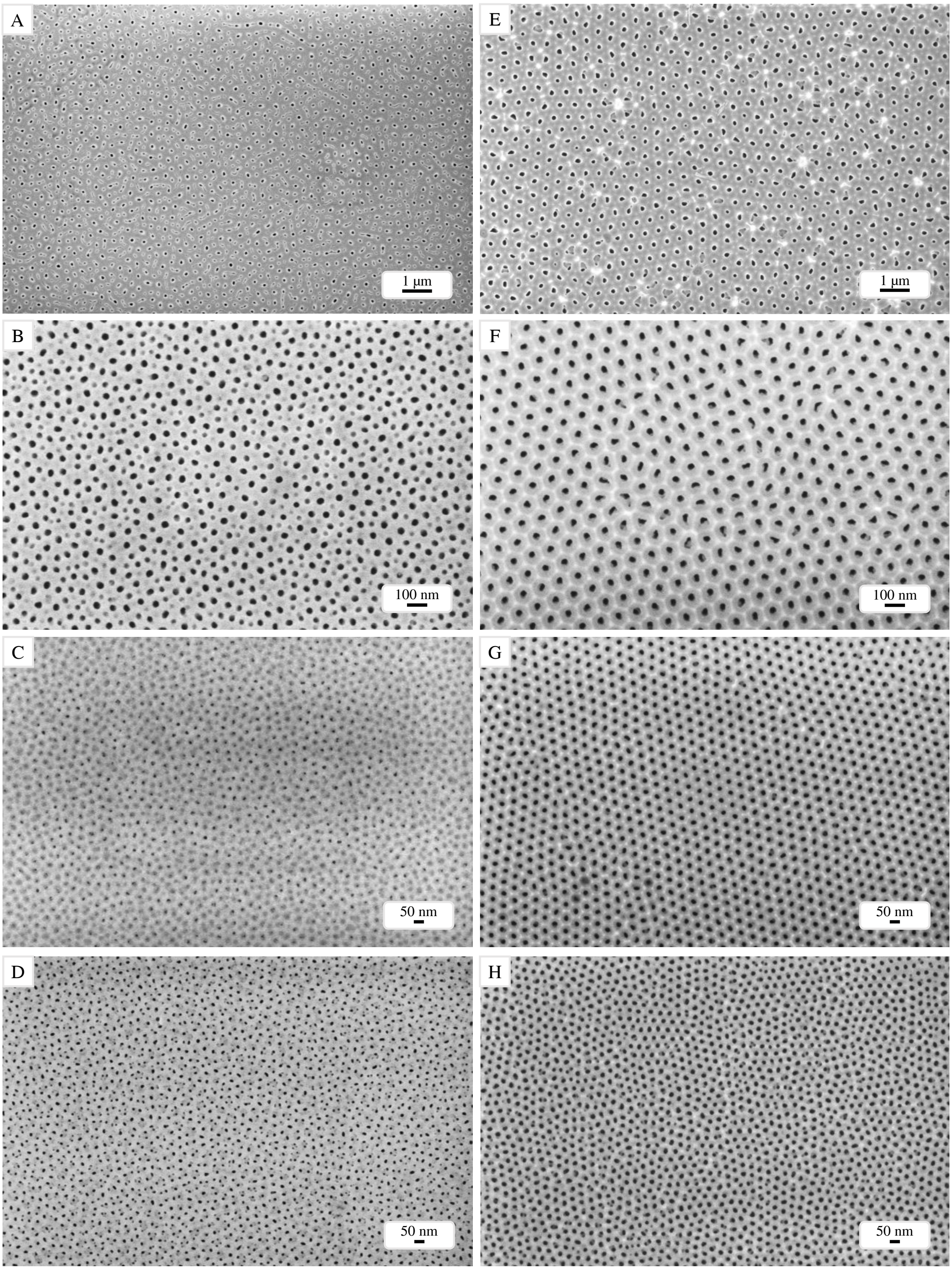

Figure S1. FE-SEM top view images of first (a, b, c, d) and second (e, f, g, h) anodization, which is carried out in (a, e) phosphoric acid, (b, f) oxalic acid, (c, g) sulphuric acid, and (d, h) ethylene glycol containing sulphuric acid.

When only the first anodization has been performed, the AAO layer surface shows a stochastic pore distribution, there is neither a clear pattern for pore locations nor formed domains, whereas a regular hexagonal arrangement of the pores, along with wide domains, are described after the second anodization. It is noteworthy that the role of the average size of the ordered domains has been frequently analysed in the literature[4-6]. Therefore, as the enlargement of the ordered domains is associated with a lower density of structural defects, which function as electron scattering centres, a two-step anodization process has been carried out in the complete set of AAO-Al samples to analyse the rest of the morphological parameters.

To study the influence of alumina thickness, several nominal values have been selected for every electrolyte: 65, 45, 25, and 12 μm. The accurate alumina thicknesses were measured by FE-SEM cross section images and are summarized in Table S1, along with the corresponding second anodization times. The D_{int} and the D_p values, which are also included in Table S1, were obtained by digital analysis of the top-view FE-SEM images.

Table S1. Second anodization time and morphological details of the different AAO-Al samples.

Electrolyte	*2nd anod. time (h)*	*t (μm)*	D_{int} *(nm)*	D_p *(nm)*	*P (%)*
Phosphoric acid + Al oxalate	4.0	11±1	463±34	122±20	6±3
	9.1	27±1	480±22	125±25	6±3
	23.7	46±2	521±81	137±15	6±4
	33.3	59±3	523±57	146±24	7±5
Oxalic acid	4.3	10±1	103±7	36±4	11±1
	8.0	24±1	104±7	37±4	12±1
	16.0	40±2	105±3	36±3	11±1
	24.0	66±3	104±3	39±6	13±2

Sulphuric acid	2.0	12±1	64±5	22±3	10±1
	6.0	24±1	69±12	24±4	11±2
	10.3	43±2	68±5	23±3	10±1
	15.5	65±3	66±4	24±2	12±1
Ethylene glycol + sulphuric acid	9.8	13±1	50±10	16±3	10±1
	22.0	27±1	47±14	18±2	14±1
	36.0	44±2	53±18	24±1	18±1
	45.4	65±3	52±23	26±2	23±2

The interpore distance depends highly on the applied voltage[7]. As these conditions stay constant for the different alumina thickness in a common electrolyte, there is one characteristic mean D_{int} for every electrolyte: 482±23 nm for phosphoric acid, 104±1 nm for oxalic acid, 67±2 nm for sulphuric acid, and 50±3 nm for ethylene glycol containing sulphuric acid.

The pore diameter is controlled by the anodization voltage[7], so it is expected to be constant in every electrolyte. However, an increment in the D_p is observed for the thicker AAO layer, which needs a longer anodization time to grow. This enhancement is due to the solubility of the alumina in the electrolytes: longer anodization time corresponds with a higher dissolving grade of the AAO in the pore wall[6]. The D_p varies between 122±20 nm and 146±24 nm in phosphoric acid, 36±4 nm and 39±6 nm in oxalic acid, 22±3 nm and 24±2 nm in sulphuric acid, and between 16±3 nm and 26±2 nm for ethylene glycol containing sulphuric acid. The statistical distribution of D_p for 12 μm-thick AAO-Al nanostructures in every electrolyte is shown in Figure S2, similar analyses have been performed for the rest of alumina thicknesses.

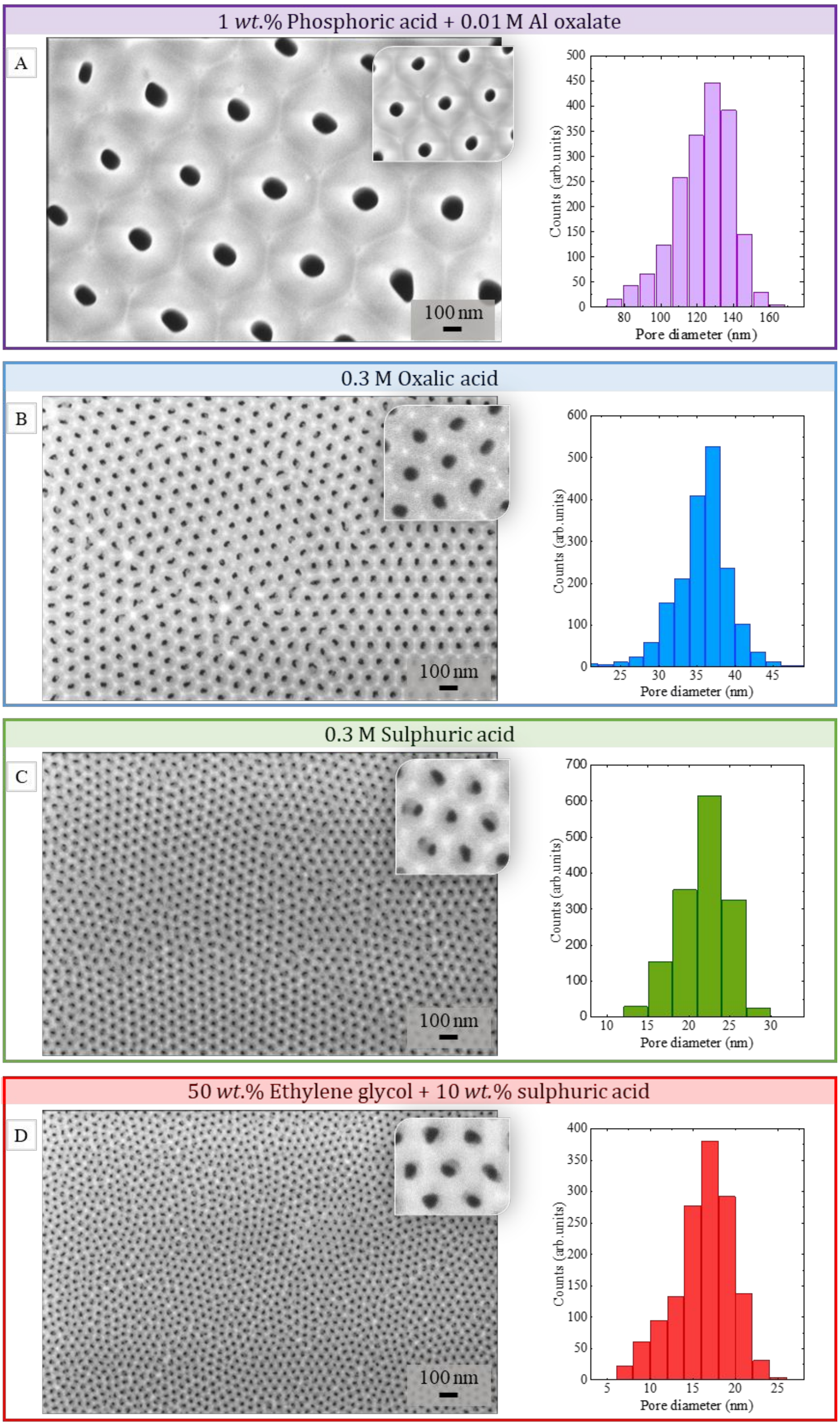
1 wt.% Phosphoric acid + 0.01 M Al oxalate
A
100 nm
Counts (arb.units)
Pore diameter (nm)
0.3 M Oxalic acid
B
100 nm
Counts (arb.units)
Pore diameter (nm)
0.3 M Sulphuric acid
C
100 nm
Counts (arb.units)
Pore diameter (nm)
50 wt.% Ethylene glycol + 10 wt.% sulphuric acid
D
100 nm
Counts (arb.units)
Pore diameter (nm)

Figure S2. FE-SEM top view images of 12 μm-thick AAO-Al samples and the pore distribution obtained in: (a) phosphoric acid, (b) oxalic acid, (c) sulphuric acid, and (d) ethylene glycol containing sulphuric acid. The hexagonal cell is enlarged in the inset.

The porosity is defined as a function of the interpore distance and the pore diameter, following:

$$P = \frac{2\pi}{\sqrt{3}}\left(\frac{D_p/2}{D_{int}}\right)^2 \quad \text{(S1)}$$

The initial porosity (P_i) of the AAO-Al samples is around 6%, 12%, and 11% for phosphoric acid, oxalic acid, and sulphuric acid, respectively. Since D_p is notably enlarged with alumina thickness in ethylene glycol containing sulphuric acid, porosity goes from 10 to 23% for 12 μm and 65 μm in thickness, respectively. The total porosity values after second anodization are shown in Table S1.

To study the influence of enhancing porosity, and consequently, enlarging AAO-Al nanostructures' D_p, chemical etching (CE) has been carried out. The maximum CE time has been limited to avoid the collapse of 12 μm–thick nanostructures for the available D_{int} values. The post-treatment FE-SEM top view images of 12 μm-thick AAO-Al nanostructures are

shown together with the pore diameters' distribution for the maximum chemical etching in Figure S3.

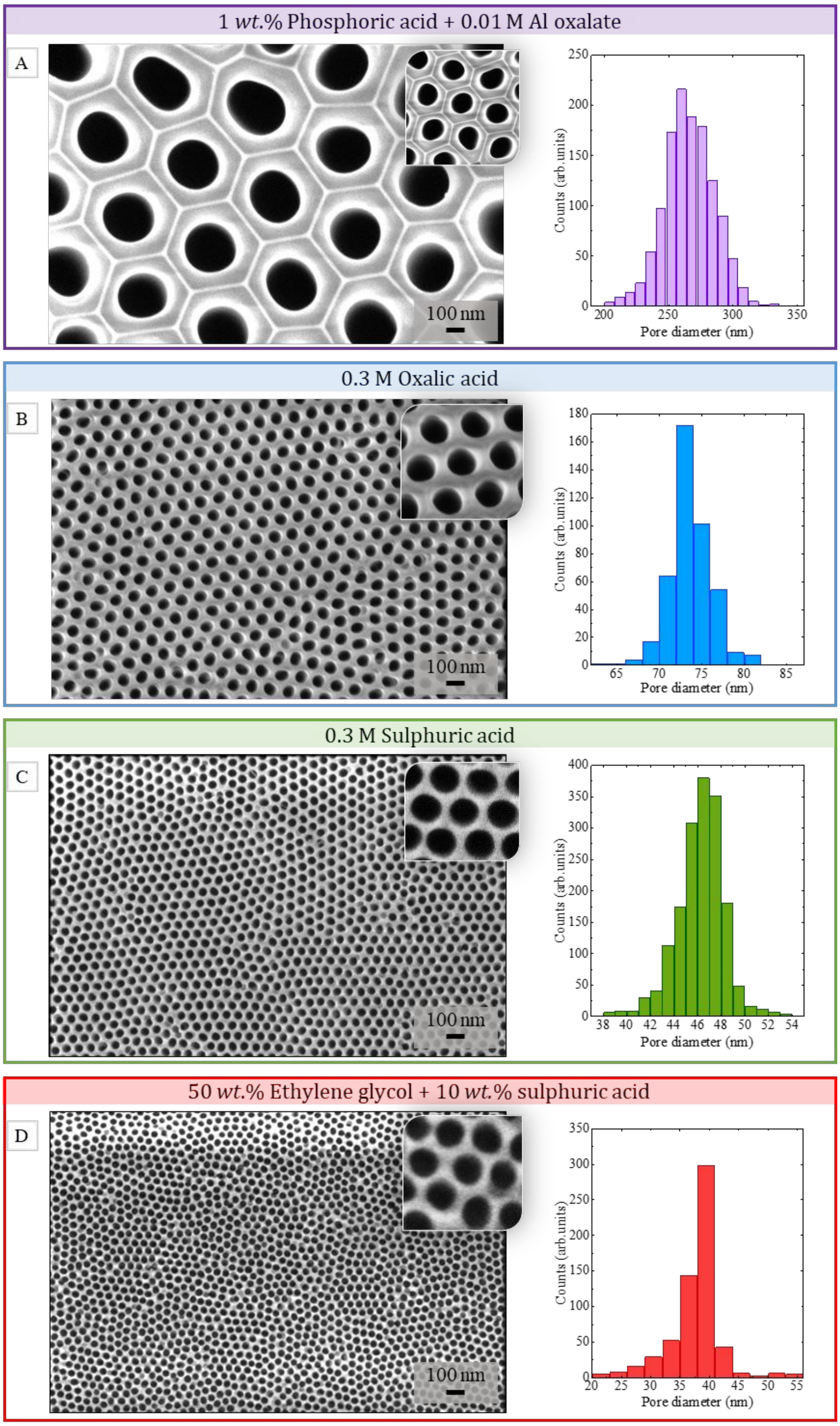

Figure S3. FE-SEM top view images of 12 μm-thick AAO-Al samples after a chemical etching and the pore distribution obtained in: (a) phosphoric acid, (b) oxalic acid, (c) sulphuric acid, and (d) ethylene glycol containing sulphuric acid. The hexagonal cell is enlarged in the inset.

The final porosity (P_f) of the AAO-Al nanostructures was found to be 30%, 46%, 43%, and 52% for phosphoric acid, oxalic acid, sulphuric acid, and ethylene glycol containing sulphuric acid, respectively, after the largest chemical etching time. The details about the evolution of the D_p with its respective P are summarized in Table S2, along with the corresponding CE time. Similar trends have been found in the other alumina thicknesses. Longer CE times could allow even higher pore diameters, etching the pore wall until collapsing the nanostructures[8], but that is not the purpose of this study.

Table S2. Evolution of the pore diameter and the porosity of AAO-Al samples with 12 μm of AAO layer due to progressive chemical etching.

Electrolyte	*t (μm)*	D_{int} *(nm)*	*CE (min)*	D_p *(nm)*	*P (%)*
Phosphoric acid + Al oxalate	11±1	463±34	0	122±20	6±3
			30	174±18	13±7
			70	225±19	21±14

			90	266±19	30±21
Oxalic acid	10±1	103±7	0	36±4	11±1
			15	52±2	23±2
			30	74±2	46±6
Sulphuric acid	12±1	64±5	0	22±3	10±1
			25	44±4	39±5
			30	46±2	43±5
Ethylene glycol + sulphuric acid	13±1	50±10	0	16±3	10±1
			6	26±4	25±3
			21	38±4	52±8

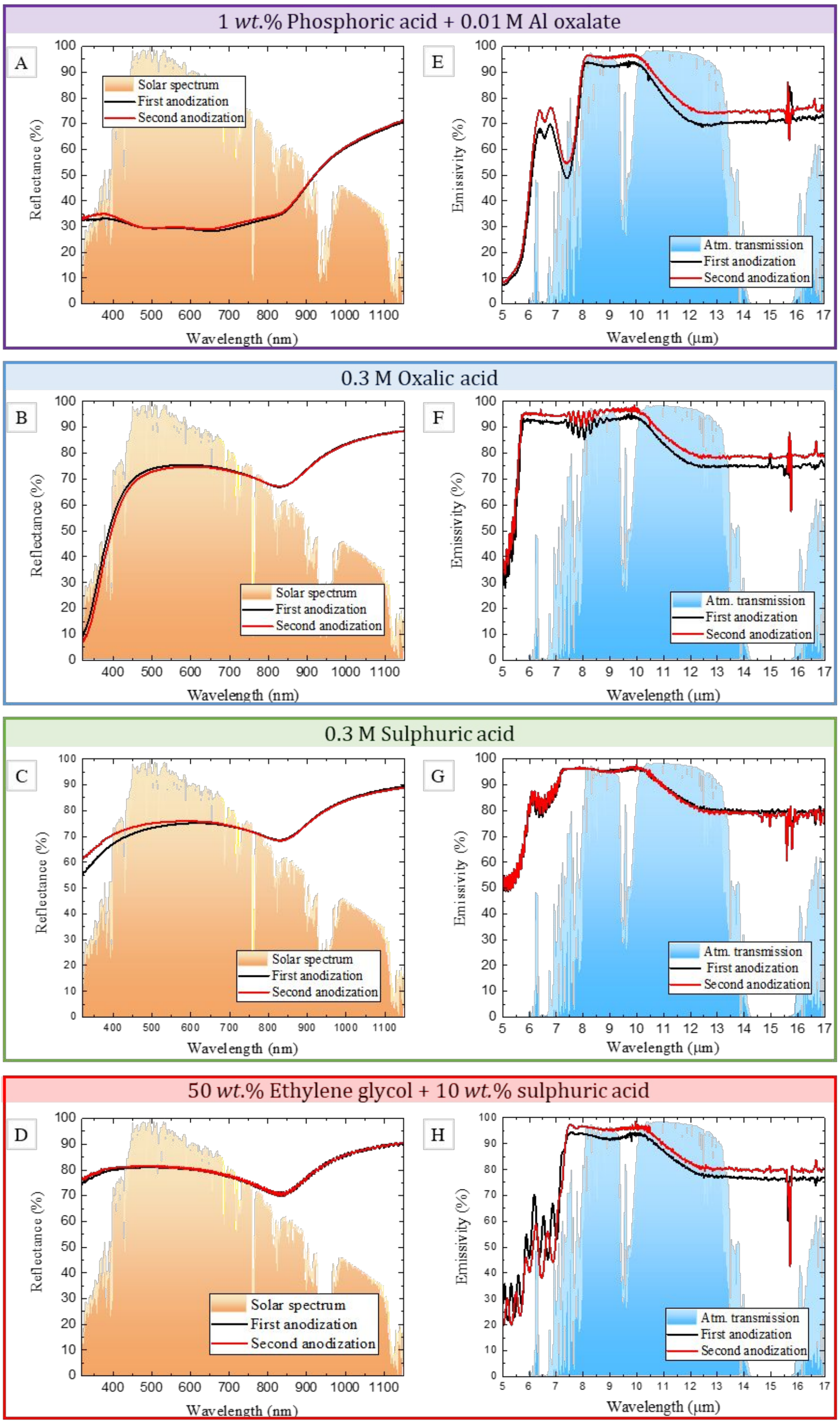

Figure S4. First and second anodization's optical characterisation, including UV-Vis-NIR reflectance measurements of AAO-Al: (a) phosphoric acid, (b) oxalic acid, (c) sulphuric acid,

and (d) ethylene glycol containing sulphuric acid, and mid-IR emissivity: (e) phosphoric acid, (f) oxalic acid, (g) sulphuric acid, and (h) ethylene glycol containing sulphuric acid.

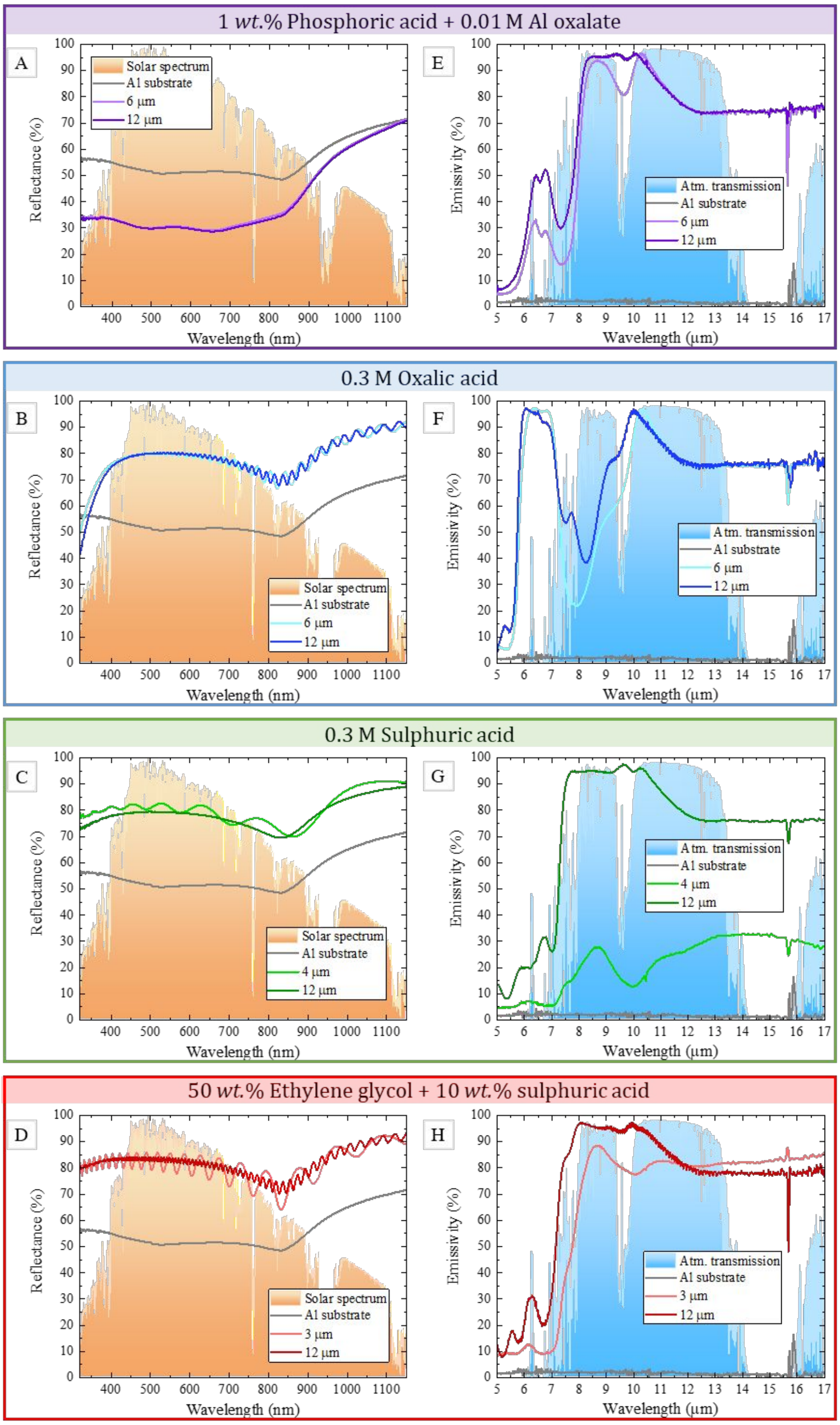

Figure S5. Characterisation of the thinnest AAO thickness case, comparing UV-Vis-NIR reflectance (a, b, c, d) and mid-IR emissivity (e, f, g, h) with AAO layer of 12 µm, and bare Al substrate in (a, e) phosphoric acid, (b, f) oxalic acid, (c, g) sulphuric acid, and (d, h) ethylene glycol containing sulphuric acid.

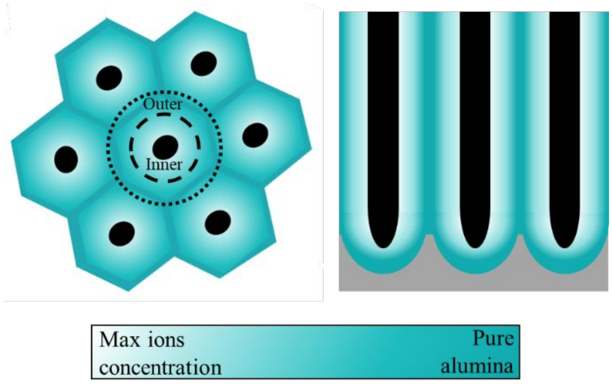

Figure S6. Scheme of hexagonal porous alumina cell: top view and cross section. Gradient illustrates the distribution of incorporated ions from the electrolyte to the alumina chemical structure.

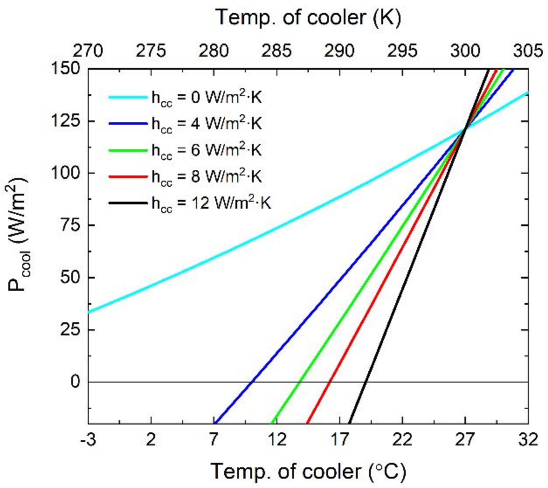

Figure S7. Cooling power *vs.* the temperature of the AAO-Al sample with 12 μm AAO layer, P_i, and anodization in sulphuric acid, considering different heat-transfer coefficients (h_{cc}).

As it is shown in Figure S8a, for the first cycle of measurements (July 4th, 2022) the temperature of the empty box on the rooftop reaches 50.6 °C during the daytime and stays at 12.7 °C during the night. The temperature of AAO-Al sample anodized on sulphuric acid is reduced to 44.5 °C during the daytime and 15.7 °C at night, which results in a higher comfort level. For the second cycle of measurements (May 20th, 2022), the temperature of AAO-Al sample fabricated on sulphuric acid is reduced from 50.6 °C to 45.5 °C during the daytime, and from 14.7 to 13.7 °C at night, which would mean preliminary steps to reduce the use of air conditioners (see Figure S8b).

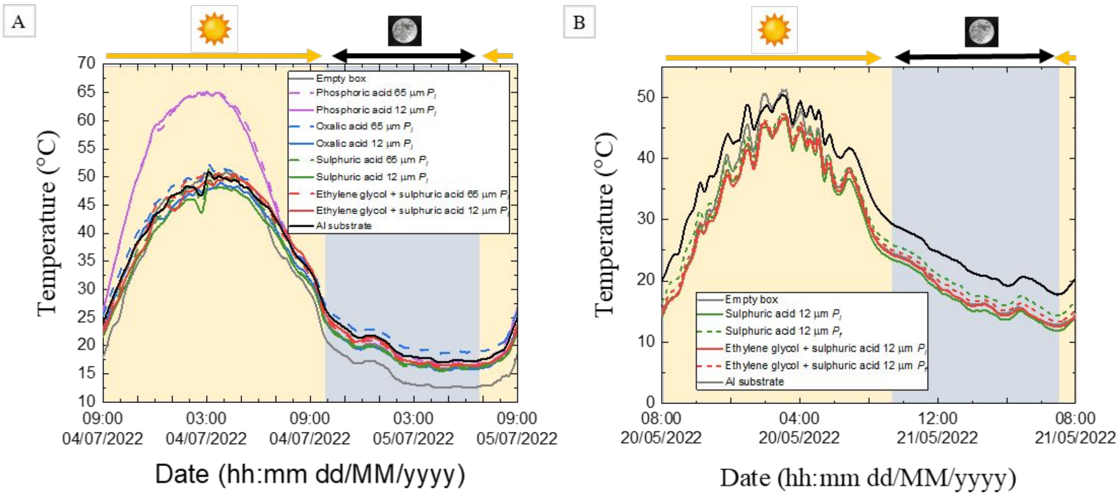

Figure S8. Temperature variations during two cycles of measurements.

Figure S9 shows that in the third cycle of measurements (August 12th, 2022) the temperature of AAO-Al sample fabricated on sulphuric acid is reduced from 58.4 °C to 50.4 °C during the daytime, while the temperature at night stays constant on a sweltering day in summer in Madrid, Spain.

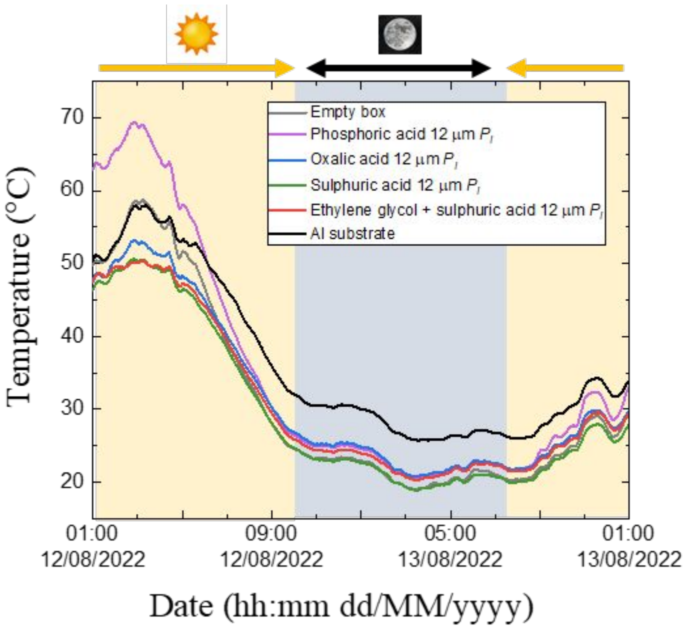

Figure S9. Temperature variations during a cooling measurement of the AAO-Al samples.

Calculations of maximum cooling power, P_{cool}, have been carried out using the experimental measurements for UV-Vis-NIR reflectance as well as the mid-IR. To evaluate P_{cool}, several contributions have been considered:

$$P_{cool}(T) = P_{rad}(T) - P_{atm}(T_{amb}) - P_{sun} - P_{cc}(T, T_{amb}) \tag{S2}$$

where T is the temperature of the AAO-Al samples, $P_{rad}(T)$ is the power radiated out, P_{atm} (T_{amb}) is the absorbed power from the atmospheric thermal radiation, P_{sun} is the absorbed power due to the solar radiation, and $P_{cc}(T, T_{amb})$ represents the parasitic loss because of non-radiative exchanges: convection and conduction.

The radiation power, $P_{rad}(T)$, emitted by the cooler is defined as:

$$P_{rad}(T) = \iint_0^{\infty} I_{BB}(T,\lambda)\epsilon(\lambda,\theta)\; d\lambda \cos\theta\; d\Omega \tag{S3}$$

where λ is the wavelength, θ the polar angle and $\int d\Omega$ the angular integral over a hemisphere. $I_{BB}(T,\lambda)$ is the spectral radiance density of a blackbody, which is defined as:

$$I_{BB}(T, \lambda) = \frac{2hc^2}{\lambda^5}\frac{1}{e^{hc/\lambda k_b T} - 1} \tag{S4}$$

where h is the Planck's constant, c is the speed of light, and k_b is the Boltzmann's constant. The thermal exchange with the atmosphere, $P_{atm}(T_{amb})$, is defined as:

$$P_{atm}(T_{amb}) = \iint_0^{\infty} I_{BB}(T,\lambda)\epsilon(\lambda,\theta)\epsilon_{atm}(\lambda,\theta)\; d\lambda \cos\theta\; d\Omega \tag{S5}$$

where $\epsilon_{atm}(\lambda,\theta) = 1 - t(\lambda)^{1/\cos\theta}$ describe the atmosphere's emissivity, and $t(\lambda)$ is the atmosphere's transmittance in the zenith direction. The absorbed solar radiation, P_{sun}, is calculated as:

$$P_{sun} = \int \epsilon(\lambda,\theta) \cos(\theta_{sun}) I_{AM1.5}(\lambda)\, d\lambda \tag{S6}$$

where $I_{AM1.5}$ is the standard AM1.5G spectrum of the solar irradiance, and θ_{sun} is the incidence angle of the solar irradiance. Non-radiative heat exchanges are included in $P_{cc}(T, T_{amb})$, which is defined as:

$$P_{cc}(T, T_{amb}) = h_{CC}(T_{amb} - T) \tag{S7}$$

where h_{CC} is the heat-transfer coefficient.

Author information

Corresponding author:

*Email: marisol.martin@csic.es

References

[1] J.T. Domagalski, E. Xifre-Perez, L.F. Marsal, Recent Advances in Nanoporous Anodic Alumina: Principles, Engineering, and Applications, Nanomaterials, 2021, https://doi.org/10.3390/nano11020430.

[2] A. Ruiz-Clavijo, O. Caballero-Calero, M. Martín-González, Revisiting anodic alumina templates: from fabrication to applications, Nanoscale 13(4) (2021) 2227-2265, https://doi.org/10.1039/D0NR07582E.

[3] H. Masuda, K. Fukuda, Ordered Metal Nanohole Arrays Made by a Two-Step Replication of Honeycomb Structures of Anodic Alumina, Science 268(5216) (1995) 1466-1468, https://doi.org/10.1126/science.268.5216.1466.
[4] F. Li, L. Zhang, R.M. Metzger, On the Growth of Highly Ordered Pores in Anodized Aluminum Oxide, Chemistry of Materials 10(9) (1998) 2470-2480, http://doi.org/10.1021/cm980163a.
[5] M. Pashchanka, S. Okeil, J.J. Schneider, Long-Range Hexagonal Pore Ordering as the Key to Controlling SERS Efficiency in Substrates Based on Porous Alumina, The Journal of Physical Chemistry C 124(47) (2020) 25931-25943, https://doi.org/10.1021/acs.jpcc.0c02761.
[6] M. Pashchanka, Conceptual Progress for Explaining and Predicting Self-Organization on Anodized Aluminum Surfaces, Nanomaterials, 2021, https://doi.org/10.3390/nano11092271.
[7] K.S. Choudhari, C.-H. Choi, S. Chidangil, S.D. George, Recent Progress in the Fabrication and Optical Properties of Nanoporous Anodic Alumina, Nanomaterials, 2022, https://doi.org/10.3390/nano12030444.
[8] Y. Zhou, Y. Liu, Y. Li, R. Jiang, W. Li, W. Zhao, R. Mao, L. Deng, P. Zhou, Flexible radiative cooling material based on amorphous alumina nanotubes, Opt. Mater. Express 10(7) (2020) 1641-1648, https://doi.org/10.1364/OME.392241.
